# Estimation of Human Center of Mass Position through the Inertial Sensors-Based Methods in Postural Tasks: An Accuracy Evaluation

**DOI:** 10.3390/s21020601

**Published:** 2021-01-16

**Authors:** Marco Germanotta, Ilaria Mileti, Ilaria Conforti, Zaccaria Del Prete, Irene Aprile, Eduardo Palermo

**Affiliations:** 1 IRCCS Fondazione Don Carlo Gnocchi ONLUS, 50143 Florence, Italy; mgermanotta@dongnocchi.it (M.G.); iaprile@dongnocchi.it (I.A.); 2 Department of Mechanical and Aerospace Engineering, Sapienza University of Rome, 00185 Roma, Italy; ilaria.conforti@uniroma1.it (I.C.); zaccaria.delprete@uniroma1.it (Z.D.P.); eduardo.palermo@uniroma1.it (E.P.)

**Keywords:** CoM displacement, IMUs, balance, posturography, human kinematic measurement

## Abstract

The estimation of the body’s center of mass (CoM) trajectory is typically obtained using force platforms, or optoelectronic systems (OS), bounding the assessment inside a laboratory setting. The use of magneto-inertial measurement units (MIMUs) allows for more ecological evaluations, and previous studies proposed methods based on either a single sensor or a sensors’ network. In this study, we compared the accuracy of two methods based on MIMUs. Body CoM was estimated during six postural tasks performed by 15 healthy subjects, using data collected by a single sensor on the pelvis (Strapdown Integration Method, SDI), and seven sensors on the pelvis and lower limbs (Biomechanical Model, BM). The accuracy of the two methods was compared in terms of RMSE and estimation of posturographic parameters, using an OS as reference. The RMSE of the SDI was lower in tasks with little or no oscillations, while the BM outperformed in tasks with greater CoM displacement. Moreover, higher correlation coefficients were obtained between the posturographic parameters obtained with the BM and the OS. Our findings showed that the estimation of CoM displacement based on MIMU was reasonably accurate, and the use of the inertial sensors network methods should be preferred to estimate the kinematic parameters.

## 1. Introduction

The assessment of the motion of human Center of Mass (CoM) is of uttermost importance in ergonomics [1,2,3], sporting [4,5,6], and clinical practice [7,8,9,10], since it contributes to the quantitative measurements of risky imbalance and postural impairments of humans.

In balanced conditions, postural control acts on body orientation through active and passive mechanisms, based on somatosensory feedback integration, in order to maintain an upright stance [11,12]. Thus, both predictable and hazardous perturbations can be properly counteracted through online corrections of CoM dynamics [13,14]. As the upright balance relies on the continuous compensation of gravity and external perturbations, the acceleration of the whole CoM is constantly varying in the three-dimensional space, to avoid accidental falls. In static conditions, falls occur when the CoM exceeds the Base of Support (BoS), defined as the area beneath the subject’s points in contact with the supporting surface [15,16]. Measuring the real-time CoM dynamics during postural steady tasks would allow monitoring the deficit of postural control and prevent unsafe conditions, as well as assess the improvement in postural control after a rehabilitation intervention.

In clinical practice, postural impairments related to aging or neuromuscular disorders can be assessed through kinematic [17,18,19] and electromyographic [20,21,22] analysis, using stereo-photogrammetry, force platforms, and electromyography (EMG) systems. However, traditional instrumentation reports numerous limits, such as the high cost, the limited workspace, and the need for skilled operator supervision. These limitations fuel the development of novel methods, involving the lowest number of sensors that guarantees the highest measurement accuracy and greatest usability. Kinematics evaluation of postural control using a single inertial sensor has the advantage of faster wearability, reducing the time consumption of the test. Moreover, it allows the assessment of postural capabilities in a wider range of ecological scenarios, such as the workplace, for biomechanical risk evaluation [23,24], in a sporting environment to enhance athlete performances [25,26,27], in a home setting for continuous monitoring of patients‘ status [28,29], and in the outpatient clinic during ambulatory assessments and follow-up visits [30,31,32]. 

In the last decade, using one inertial sensor placed at the level of the L5 vertebra was demonstrated to provide sensitive and reliable measurements for postural control assessment [33]. In this context, several methods were investigated for the measurements of human CoM displacement in three-dimensional space, involving one or more inertial sensors. According to the literature, two prevalent methods were investigated in the last years for measurement of human CoM dynamics—the strapdown integration and the inertial sensors network. In most cases, the first method relies on one sensor placed at the level of the L5 lumbar vertebra, while the inertial sensors networks are based on a biomechanical model with more than two sensors placed on the human kinematic chain.

For the estimation of CoM dynamics during walking tasks, through the use of an inertial sensor placed on the pelvis, a strapdown integration method was used in the study by Floor et al. [34]. The main idea behind this approach is that displacement in the three-dimensional space of a body can be obtained through the double integration of the accelerometer signal of a sensor placed on the body itself. To reduce the drift phenomenon due to the integration process, a drift correction was computed by detrending the integrated signal for each gait stride, with the best linear fit. A similar approach was applied in a study by Reenalda et al. for the estimation of CoM dynamics in running marathons [6]. In 2020, Cardarelli et al. suggested a magnetometer-free approach for the estimation of CoM human displacement and orientation, based on the strapdown integration method [35]. Since magnetic disturbances are a common phenomenon that can affect sensor orientation and increase the drift phenomenon, an orientation method with a variant of the Kalman filter, without magnetometer data, was adopted. Drift was also compensated with restarting integration every left–right gait cycle during treadmill walking. 

Recently, some algorithms based on an inertial sensor network were proposed for the estimation of the whole CoM dynamics, the latter being estimated through a weighted average of the CoM position of each human body segment in three-dimensional space. As an example, a similar approach was tested by Fasel et al. in alpine ski racing [36]. The authors compared two models based on sensor networks for the assessment of the CoM displacement of the ski athlete—a full 3D model; and a simplified model, based on head and sternum segments only. Many other authors evaluated the accuracy of the CoM position provided by the marketed MIMU systems and software [37], like Pavei et al. [38], or Guo et al. [39], expressing various levels of satisfaction in the performance. However, the algorithm used by the system for calculating the CoM trajectory was not explained in details [40], and thus it would be difficult to replicate it in an embedded solution for the auspicable real-time COM evaluation.

Recently, a biomechanical model based on the lower limb kinematic chain was developed by Guaitolini et al. in 2019 [41]. In this study, the inertial sensor network approach was used for the ambulatory assessment of human CoM trajectory, with respect to the foot, in the stance phase, during walking tasks. However, anthropometric measurements and sensor-to-segment calibration were performed through an OS, and the performance evaluation was cleansed of these two important sources of error, and still not reproduced through a fully wearable solution.

Despite the high interest in the use of wearable sensors for the measurements of human CoM dynamics, in most literature cases, the CoM assessment with inertial sensors was computed during dynamic tasks such as walking, running, or skiing, in which the CoM dynamics assumed large fluctuations in the 3D space [6,34,35,37,38,41,42]. Only a few studies verified the accuracy of IMU-based methods for the measurement of CoM trajectories during standing tasks [43,44]. In postural tasks, the assessment of the 3D CoM trajectory with inertial sensors could be more demanding. In these cases, the CoM dynamic assumes smaller oscillations, leading to higher errors in its estimation because of the drift error. Moreover, in the analysis of CoM dynamics during steady and postural tasks, it is not possible to correct the drift phenomenon with zero-velocity updates approach or the integration-restart method. In this scenario, the use of an inertial sensors network method with a smaller number of sensors could represent a trade-off between accuracy and feasibility of use.

The present study aimed at analyzing the implications of using an inertial sensor network method to assess CoM dynamics, in comparison to a reduced set-up involving strapdown integration in steady and postural tasks involving increasing CoM sways. A comparative analysis between the strapdown approach based on one sensor-worn, and the inertial sensor network method involving seven MIMUs placed on lower limbs, was conducted to assess the best performing measurement approach for 3D CoM dynamics in postural tasks.

## 2. Materials and Methods

### 2.1. Subjects

A cohort of fifteen healthy subjects (nine males and six females, mean age 27.7 ± 5.3, mean body mass 67.3 ± 9.3 kg, and mean height 171.9 ± 6.5 cm) was enrolled in this study. All participants were able to stand independently without aids. Subjects with cognitive, vestibular, or visual deficits, neuromuscular diseases, orthopedic, or neurological surgery interventions in the last three years, were excluded from the experimental session. All participants gave written consent before being included in the experimental session. The protocol was designed and conducted in accordance with the Ethical Standard of the 1964 Declaration of Helsinki and were approved by the Institutional Ethics Committee of the IRCCS Fondazione Don Carlo Gnocchi (FDG_17.4.19).

### 2.2. Experimental Setup

Seven wireless Inertial Measurement Units (MIMUs MTw, Xsens Technologies - NL), including a 3-axes accelerometer (± 160 m/s^2^ full scale (FS)), a 3-axes gyroscope (± 1200 °/s FS), and a 3-axes magnetometer (± 1.5 Gauss FS) were used to gather kinematic data of the lower body segments of each subject. More specifically MIMUs were placed—(i) posteriorly on the pelvis on the median sacral crest and just below the anterior sacral promontory, (ii) on the mid-thighs between the greater trochanter and the lateral epicondyle, (iii) on the mid-shanks between the lateral condyle and the malleolus, and (iv) on the instep of the feet. Elastic tapes were used to attach each sensor to the body segment and limit relative movements between them. The sampling rate was set at 40 Hz. An 8-camera optoelectronic system (OS, SMART D500, BTS, Milan, Italy), with an accuracy in the estimation of marker trajectories of less than 1 mm, was used as the reference measurement system. The sampling rate was set at 200 Hz for the OS. Each subject was instrumented with four reflective markers placed on the anterior and posterior iliac spines, according to the Plug-In-Gait model [45]. In post-processing, marker trajectories gathered from the OS were down-sampled at 40 Hz, to match the frequencies.

**Figure 1:**
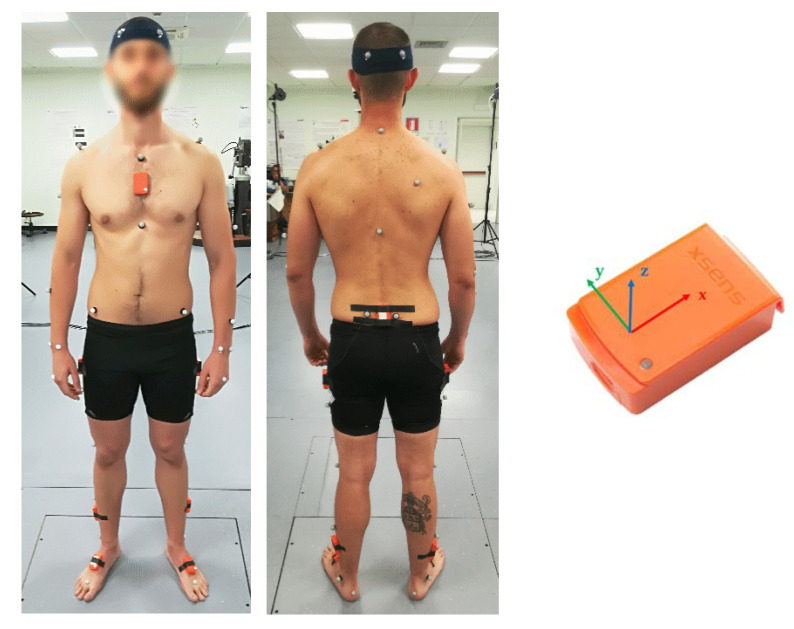
MIMUs and markers position on one subject as well as the inertial sensor reference system. Only the MIMUs placed on the pelvis and the lower limbs and the markers placed on the pelvis were considered in this study.

To guarantee consistent sensor and marker location on the body segment, the same expert operator instrumented all participants. The two systems were simultaneously triggered at the beginning of each acquisition. More specifically, a BNC cable was used to provide an external trigger, i.e., a square signal ranging from 0 to +5 V, to the MIMUs Awinda Station, through the motion capture system. In Figure 1, the sensors’ and markers’ position is shown on one subject.

### 2.3. Experimental Procedure

**Figure 2:**
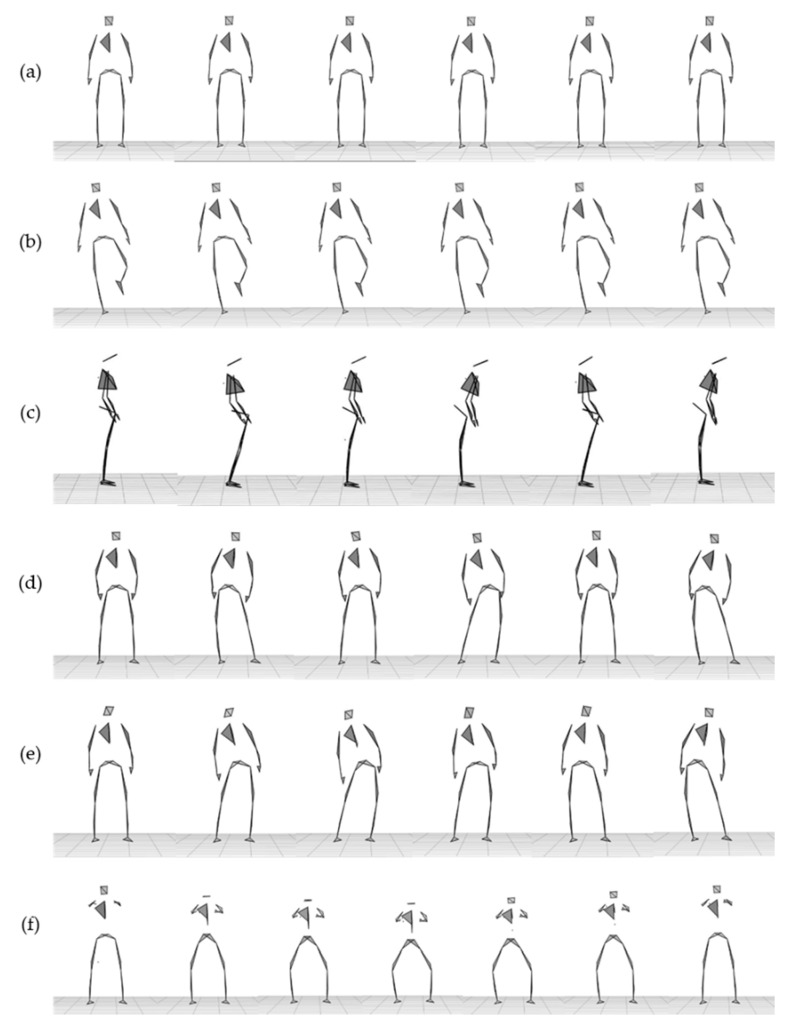
The overall marker-set tracked by the OS of a healthy subject performing the experimental protocol. (**a**) Double Leg Stance task, (**b**) Single Leg Stance task, (**c**) Anterior–Posterior (AP) sway task, (**d**) Mediolateral (ML) sway task, (**e**) Free sway task, and (**f**) Squat task.

Before each session, all tested subjects were asked to perform a Functional Calibration (FC) procedure advised by an operator. The FC procedure provided sensor orientations with respect to the body segment, to ensure the body-to-sensor alignment [46]. It consisted of a standing and sitting task, each lasting 5 s. Afterward, all subjects stood in a comfortable upright bipedal position, on the center of the calibration volume of the OS. All subjects were asked to wear comfortable gym clothes and perform the experimental session barefoot. The experimental session consisted of six tasks—(i) standing still in the double-leg stance (”Double Leg Stance task”); (ii) standing still in the right-leg stance (“Single Leg Stance task”); (iii) standing in the double-leg stance swinging pelvis body segment in the anteroposterior direction (”AP sway task”) and (iv) in the mediolateral direction (”ML sway task”); (v) standing in the double-leg stance performing a free pelvis oscillation (”Free sway task” ); and (vi) performing a squat (“Squat task”). The monopodalic task, Single Leg Stance task, was performed with the right-leg stance only, as only healthy young subjects without motor asymmetry were considered in this study. Figure 2 shows the overall marker-set tracked by the OS of a healthy subject performing the experimental protocol.

Each task was performed twice, and the order of the tasks was randomized. For each participant, the following anthropometrics were gathered by the same expert operator—(i) pelvis width (w_pl_) as the distance between right and left anterior iliac spines; (ii) pelvis height (h_rpl,_ h_lpl_) as the distance between the horizontal line passing through the right/left anterior iliac spine and the right/left greater trochanter; (ii) thigh length (l_rth,_ l_lth,_) as the distance between the right/left greater trochanter and the right/left flex-extension knee joint axis; (iii) the shank length (l_rsh,_ l_lsh_) as the distance between the right/left flex-extension knee joint axis and the right/left lateral malleolus; (iv) foot height (h_rft,_ h_lft_) as the distance between the right/left lateral malleolus and the ground; and (v) the foot length (l_rft,_ l_lft_) as the distance between the vertical line passing through the right/left malleolus and the 5th right/left toe proximal phalanx. Each subject was asked to perform a series of squats for the identification of the flex-extension knee joint axis. All anthropometrics were measured for both the right and left sides.

### 2.4. Data Processing

All data were analyzed off-line, using the MATLAB (v.2015b, MathWorks, Natick, MA, USA) program. Two different methods were used to estimate the CoM in the static and dynamic postural tasks.

#### 2.4.1. First Method: Inertial Sensor Network

**Figure 3:**
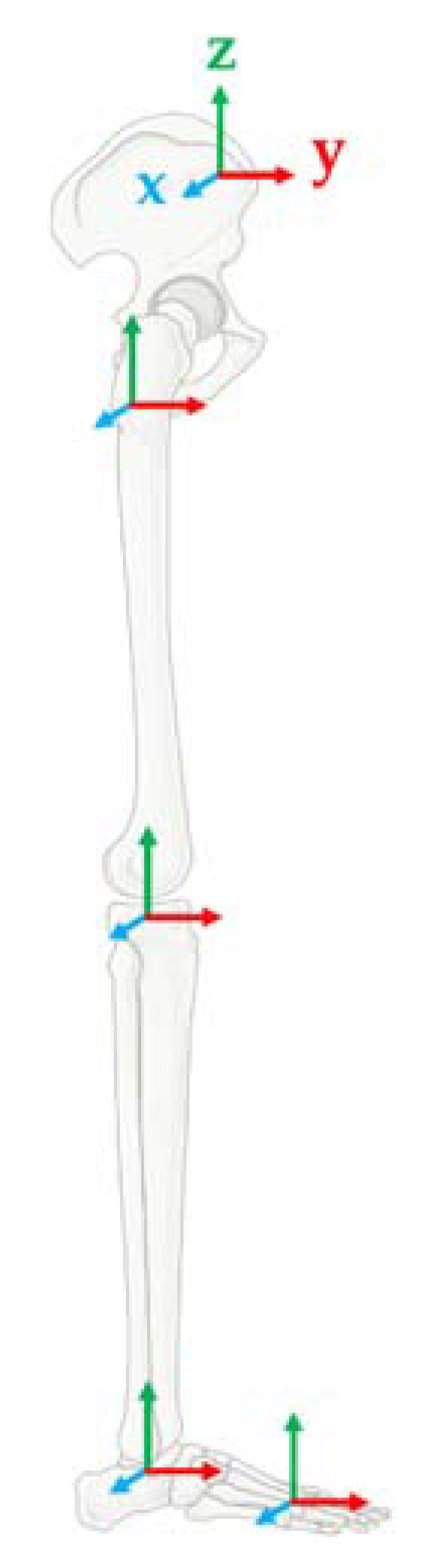
Reference systems for the pelvis body and the lower limb.

The first method concerned the development of a Biomechanical Model (BM) composed of nine body segments based on an inertial sensors network of seven MIMU [41]. Similar to [47,48], a kinematic chain composed of nine inertial body segments was adopted for the BM. More specifically (i) pelvis (pl), (ii–iii) right and left thighs (rth, lft), (iv–v) right and left shanks (rsh, lsh), (vi–vii) right and left hindfoot (rhft, lhft), and (viii–ix) right and left forefoot (rfft, lfft). A reference body frame was defined for each body segment as follows—*z*-axis vertically directed and pointing upward, yz-plane parallel to the sagittal plane, and *y*-axis as the anterior/posterior one, pointing forward, as reported in Figure 3.

According to [46], the rotation matrix between two adjacent body segments was computed as follows:

(1) RbIbJ=(RgndsIRsIbI)TRgndsJRsJbJ

where b_I_ and b_J_ are the proximal and distal body segment and the RgndsI is the quaternion-derived rotation matrix representing the I-sensor orientation, i.e., the relative rotation between the s_I_ and the ground reference system (gnd):

(2) $$R\;=\;\left[\begin{array}{ccc} 1-2s(q_{j}^{2}+q_{k}^{2}) & 2s(q_{i}^{}q_{j}^{}-q_{k}^{}q_{r}^{}) & 2s(q_{i}^{}q_{k}^{}+q_{j}^{}q_{r}^{})\\ 2s(q_{i}^{}q_{j}^{}+q_{k}^{}q_{r}^{}) & 1-2s(q_{i}^{2}+q_{k}^{2}) & 2s(q_{j}^{}q_{k}^{}-q_{i}^{}q_{r}^{})\\ 2s(q_{i}^{}q_{k}^{}-q_{j}^{}q_{r}^{}) & 2s(q_{j}^{}q_{k}^{}+q_{i}^{}q_{r}^{}) & 1-2s(q_{i}^{2}+q_{j}^{2}) \end{array}\right]$$

(3) $$q=q_{r}+q_{i}i+q_{j}j+q_{k}k$$

where in case of the unit quaternion, s=1, otherwise $s={\|q\|}^{-2}$. More specifically, the orientation of each IMU was computed with a Kalman filter for 3 degrees-of-freedom orientation, called XKF-3w. The rotation and the velocity increments computed through a strap-down integration algorithm [49,50], along with the magnetometer samples, were used to compute the 3D sensors’ orientation. More details of the XKF-3w are reported in [51]. The RsIbI represents the rotation matrix between the I-th body segment and the I-th sensor obtained through the functional calibration procedure. More details on the FC procedure are reported in [46]. 

The roto-translation matrix between two adjacent body segments was obtained by considering the following equation:

(4) TbIbJ=[RbIbJobIbI,bJ0001]

where obIbI,bJ is the origin of the distal segment frame in the proximal segment. Each origin was set in accordance with the anthropometrics of the body segment, as reported in the following equations:

(5) oplpl,rth=[wpl200]T

(6) oplpl,lth=[−wpl200]T

(7) orthrth,rsh=[00−(hrpl+lrth)]T

(8) olthlth,lsh=[00−(hlpl+llth)]T

(9) orshrsh,rhft=[00−lrsh]T

(10) olshlsh,lhft=[00−llsh]T

(11) orhftrhft,rfft=[0lrft−hrft]T

(12) olhftlhft,lfft=[0llft−hlft]T

After obtaining the roto-translation matrices for each body segment, the roto-translation matrix between the right forefoot and the pelvis body segment and its transpose were obtained as follows:

(13) Tplrfft=TplrthTrthrshTrshrhftTrhftrfft

(14) Tplrfft=[Rplrfftoplpl,rfft0001]

(15) Trfftpl=[(Rplrfft)T−(Rplrfft)Toplpl,rfft0001]

Finally, the CoM position was computed as the origin of the pelvis body segment in the right (or left) forefoot frame:

(16) CroMBM=orfftrfft,pl=−(Rplrfft)Toplpl,rfft

Similar consideration was applied to the left side. Thus, the estimation of the human center of mass through the use of the BM was computed, both on the right (CroMBM) and the left (CloMBM) side of the human kinematics chain. In this study, data provided only by the right side was used. To match the components, the CoM displacement obtained through BM was then rotated in the OS coordinate system.

#### 2.4.2. Second Method: Strapdown Integration

Similarly to [34], the second method was based on the strapdown integration (SDI) of the acceleration signal of the sensor placed on the pelvis body. The rotation matrix reported in Equation (2) was used for the component change of the acceleration signals from the sensor frame (as) to the ground reference system:

(17) agnd= Rgnds× as

After the component change, the gravitational contribution was removed and the acceleration signal in the global frame was integrated in a straightforward. A high-pass first-order Butterworth filter with a cut-off frequency of 0.2 Hz was used for the anterior–posterior (AP) and mediolateral (ML) components, while a cut-off of 0.5 Hz was adopted for the vertical (V) component of the velocity signal, as reported in [34]. The displacement of the pelvis body segment was obtained with a second straightforward integration and filtering [34]. Thus, the center of mass of the human body from the strapdown method ($CoM_{SDI}$) was computed as the three-dimensional components of the pelvis displacement.

For the sake of clarity, for both methods, the ground reference system was defined according to the OS system; the *x*-axis along the direction of progression, as the anterior/posterior axis pointing forward, the *y*-axis vertically directed pointing upward and the *z*-axis completing a right-handed coordinate system. The sensors reference system is reported in Figure 1.

**Figure 4:**
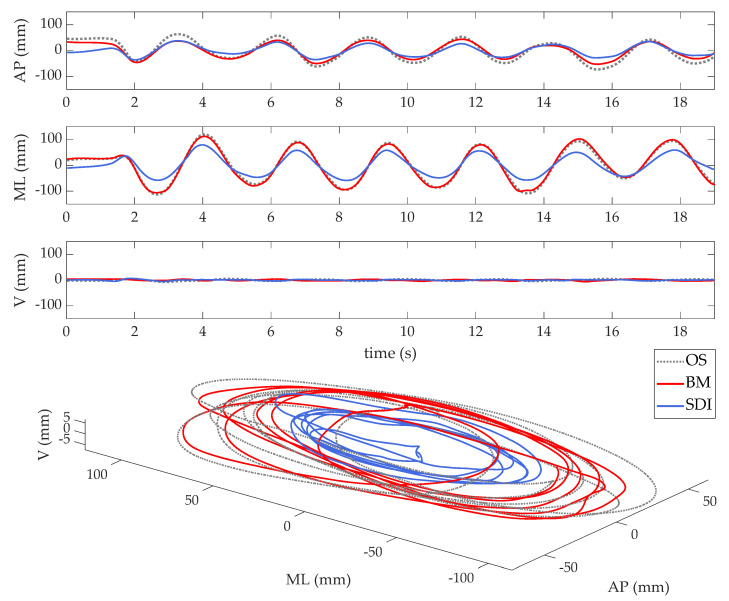
CoM trajectory of a Free Sway task (anteroposterior, AP; mediolateral, ML; and vertical, V, directions) performed by a representative subject, as obtained through the Biomechanical Model (BM, red) and the Strapdown Integration Method (SDI, blue), as compared to the gold standard (the optoelectronic system, OS, dotted black line).

The reference CoM displacement was obtained through OS as the average of the markers’ trajectories of the iliac spines in the OS coordinate system [4]. Before computing the CoM trajectories, all raw data were filtered with a 5th order Butterworth low-pass filter, with a cut-off frequency of 10 Hz. The time course of the CoM trajectories provided by the OS and the two MIMU-based methods in a representative subject is reported in Figure 4.

The root mean square error (RMSE) between the OS and each MIMU-based trajectory was computed on the modulus (mod) and each component of CoM—anteroposterior (AP), mediolateral (ML), and vertical (V), and used to assess the accuracy of the SDI and the BM [52].

In addition, the kinematics parameters commonly used in postural evaluations [10], such as (a) the range of motion of CoM displacement in the AP (AP Sway) and ML (ML Sway) directions, (b) the total path length in the AP and the ML plane, divided by the task duration (Mean Sway Velocity), and (c) the 95% confidence ellipse area (95% Sway Area) were computed for all methods. For the kinematic parameters, the accuracy of the methods was estimated by considering the following error:

(18) $$e_{r}=\;x_{i}-x_{OS}$$

where x_i_ is the kinematic parameter obtained with BM or SDI and x_OS_ is the corresponding reference value obtained with OS.

### 2.5. Statistical Analysis

Statistical analysis was performed with the SPSS package version 25 (IBM-SPSS Inc. Armonk, NY, USA). We had two repetitions available for each subject, for a total number of thirty observations for each trial. All data were tested for normality through the Shapiro-Wilk test. Paired *t*-tests were used to compare the RMSE in the estimation of the CoM trajectories of the two MIMU-based methods, as well as the errors introduced by the two analyzed methods in the estimation of the kinematic postural parameters, separately for each investigated trial. Finally, Pearson’s correlation coefficient was used to investigate the correlation between the kinematic parameters computed either through the method based on the MIMU Pearson’s correlation coefficient was used to investigate the correlation between the kinematic parameters computed through OS and the kinematic parameters computed through the methods based on the MIMU. The coefficient values were interpreted as follows [53]—0.0–0.2 little if any; 0.2–0.4 weak; 0.4–0.7 moderate; 0.7–1.0 strong. Statistical significance was set at p < 0.05. 

## 3. Results

**Figure 5:**
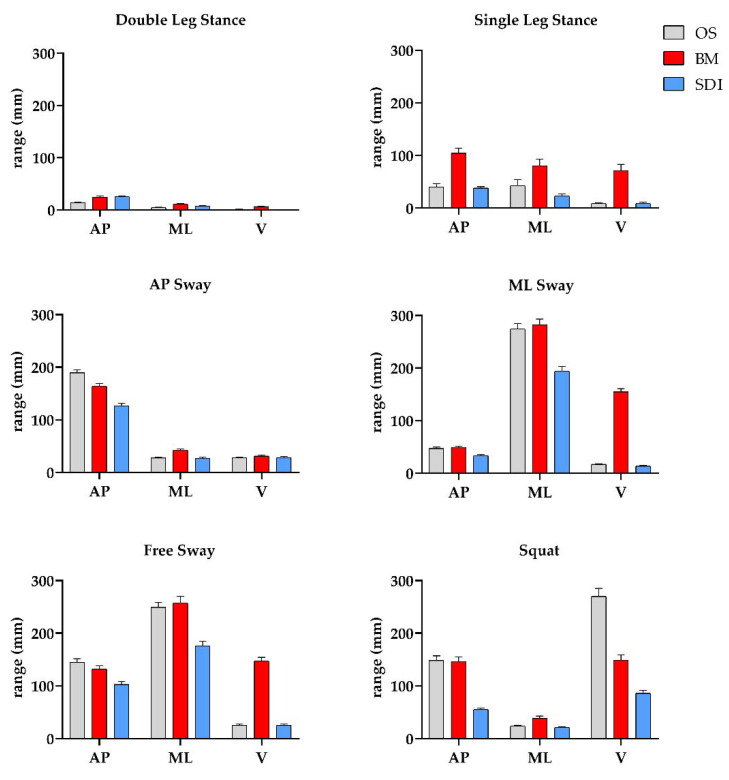
Means and 95% confidence intervals values of the range of the trajectory made by the CoM, as measured by the optoelectronic system, and estimated by the two IMU-based methods, in the anteroposterior (AP), mediolateral (ML), and vertical (V) directions, for the 6 considered tasks.

In Figure 5, the mean ranges of the oscillation of the CoM measured by the OS and estimated by both MIMU-based methods were depicted. For the inertial sensors network method, only results of the right side were reported (CroMBM), since the results of the two sides were not statistically different. As expected, the OS measurements report lower oscillations in the Double Leg Stance task in all directions, as compared to other motion conditions; moreover, the oscillation ranges in the AP, ML, and V directions were higher in the AP Sway, ML, Sway, and Squat task, respectively.

According to the OS measurement, in the Double Leg Stance, the mean values were 13.4 ± 7.5 mm (AP), 4.5 ± 3.5 mm (ML), and 1.2 ± 0.8 mm (V). For the AP, ML, and V directions, the highest values were 189.7 ± 28.2 mm in the AP Sway task, 274.3 ± 57.1 mm in the ML Sway task, and 269.8 ± 83.5 mm in the Squat task. As expected, comparing the Double Leg Stance and the Single Leg task, higher values of the oscillations’ range was found in the Single Leg task in all directions—in the monopodalic stance, a greater CoM dynamics was required to maintain an upright position as a consequence of a reduced base of support.

### 3.1. Comparison of the Root Mean Square Errors of the Investigated Methods

In Table 1, descriptive statistics of the RMSE related to the SDI and the BM methods, as well as their difference and the results of the statistical analysis are reported, separately for the four components and the six investigated trials.

With respect to the SDI, considering the three directions, the mean RMSE ranged from 0.3 mm (vertical component, Double Leg Stance task) to 79.5 mm (V component, Squat task); instead, the lower mean RMSE value of the BM was 0.9 mm (V component, Double Leg Stance task), while the higher was 26.2 mm (AP component, Single Leg Stance task). 

In all but the AP component, the modulus was in the Double Leg Stance trial and the ML component in the Single Leg Stance trial. Specifically, the RMSE of the AP component was always lower for the BM methods, except for the Single Leg stance task. The ML component was lower for the SDI in the Double and Single Leg Stance, in the AP Sway and the Squat; the RMSE of the V component was always lower for the SDI method, except for the Squat task. Finally, the RMSE of the modulus was always lower for the BM method, except for the Single Stance Method. Considering the three directions, the highest differences favorable to the SDI was 15.2 mm (AP component, Single Leg Stance task), the highest favorable to the BM was 55.1 mm (vertical component—Squat task).

### 3.2. Comparison of the Errors Introduced in the Estimation of Postural Variables

**Figure 6:**
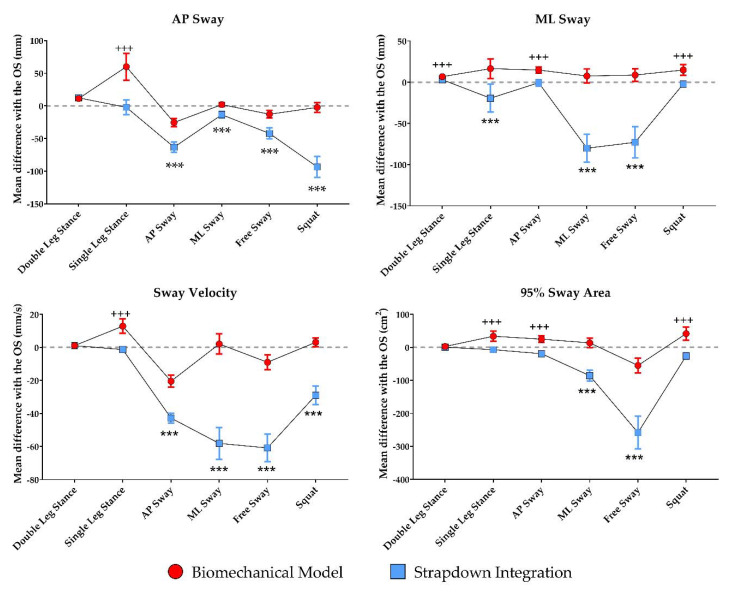
Errors (means and 95% Confidence Intervals) in the estimation of the analyzed kinematic parameters made by the Biomechanical Model and the Strapdown Integration method, using the Optoelectronic System (OS) as the reference, for each investigated task. The symbols indicate a statistically significant difference (p < 0.001) between the two methods when a higher absolute error is associated with the Biomechanical Model (+++) or the Strapdown Integration method (***).

In Figure 6, the errors in the estimates of the analyzed postural variables, as well as their difference and the results of the statistical analysis are depicted, separately for the six investigated trials.

All but the AP Sway, the Mean Sway velocity, and the 95% Sway Area in the Double Leg Stance task were different between the two methods. The error in the AP Sway was lower using the SDI method in Single Leg Stance and using the BM for the four remaining tasks. The ML Sway was lower for the SDI methods in the Double Leg Stance, AP sway, and Squat tasks, and lower for the BM in the Single Leg Stance, ML Sway, and Free Sway task. The SDI provided a better estimation of the Sway Velocity in the Single Leg Stance task, while the BM provided better results in the AP Sway, ML Sway, and Free Sway tasks, as well as in the Squat task. Finally, a better estimate of the 95% Sway Area was provided by the SDI in the Double and Single Leg Stance, AP Sway, and Squat tasks, while better performance was obtained with the BM in the ML Sway and the Free Sway tasks. 

### 3.3. Correlation Analysis

Pearson’s correlation coefficients between the kinematic parameters computed using either method, based on the inertial sensors and the OS are reported in Table 2. Considering the BM method, 18 out of 24 analyzed parameters (4 parameters, 6 different tasks) showed a strong correlation with those computed through the OS, while among the parameters computed according to the SDI, only 8 achieved a Pearson’s coefficient higher than 0.7. For the BM method, the Pearson’s correlation coefficients ranged from 0.331 (95% Sway Area, Double Leg Stance task) to 0.980 (Mean Sway Velocity—Free Sway task). For the SDI method, the range was from 0.154 (95% Sway Area, Double Leg Stance task) to 0.962 (Mean Sway Velocity—AP Sway task).

## 4. Discussion

In this work we compared the accuracy of two MIMUs-based methodologies, to estimate the three-dimensional CoM position during postural tasks involving CoM displacements of different amplitude and direction. Specifically, one method was based on an Inertial Sensors Network, with seven sensors, while the other involved a strapdown integration of the signal provided by a single sensor, placed on the pelvis. An OS-based method was used as a gold standard. The accuracy was determined both in terms of RMSE between each method based on MIMUs and the gold standard, as well as in terms of the absolute error in the estimation of kinematic parameters commonly used as hallmarks of the postural control.

### 4.1. Accuracy of CoM Displacement Estimation

Overall, the mean RMSE was always lower than 26 mm for the BM, and 85 mm for the SDI. Considering the first method, our results were comparable to those obtained by Guaitolini et al. [41] during treadmill gait, using an inertial sensor network composed of 7 IMUs, or Najafi et al. [27], using two or three sensors to estimate the trajectory of the CoM. More specifically, our methodology overcomes the limitation of the work of Guaitolini et al. [41], as in our study, the anthropometrical measures were taken by means of a measuring tape, instead of through the OS system. This, in addition to the use of a functional calibration procedure for the sensor-to-segment rotation estimation, allowed us to present and validate a procedure that is completely performable out of the laboratory setting. However, because of the different analyzed tasks (treadmill walking vs. postural tasks), a direct comparison could not be performed.

In our study, we analyzed tasks with different amplitudes of CoM excursion, both lower and higher than those normally achieved during a walking task, especially on a treadmill. For example, Floor et al. [34] found mean excursions of 1.9 ± 0.4 cm, 2.2 ± 0.7 cm, and 3.5 ± 0.3 cm in the AP, ML, and V direction, respectively. High excursions of the vertical distance between CoM and the base of support, similar to those observed in the Squat task reported in our study, were presented in the study by Fasel et al. [34], during the skiing tasks. In this context, higher accuracy was found in [34], comparing tasks with similar oscillation ranges. Noteworthily, the kinematic chain adopted in the study of Fasel et al., involves a higher number of inertial sensors placed both on the lower than on the upper body segments. This could suggest the need for deeper investigation of the granularity of the Inertial Sensors Network, in the estimation of CoM trajectory during postural tasks with high CoM excursions.

Considering the SDI methods, in tasks with similar oscillation, our results were comparable to those obtained by Floor et al. [34] and Cardarelli et al. [35], but higher errors were found when compared to those by Myklebust et al. [5].

Comparing the two methods, a better performance was achieved by the SDI in the two most static tasks, i.e., the Double Leg Stance and the Single Leg Stance, or in the directions with a small displacement amplitude (as in the ML direction for the AP Sway task, or the vertical direction for all tasks but the Squat). On the contrary, lower RMSE values were obtained with the BM in tasks or directions with higher displacement. It is worth mentioning that the highest difference in terms of RMSE in favor of the SDI method was 1.5 cm (Single Leg Stance task, AP direction), while the highest difference in terms of RMSE in favor of the BM method was 6.2 cm (Squat task, modulus). Moreover, our sample was composed of healthy young men and women, who were still expected to be able to maintain the CoM during both a Double or Single Leg Stance task. On the contrary, higher oscillations in a Double Leg Stance task were expected in different samples (as neurological or orthopedic patients), which could also lead to better results with the BM methods in a static test. 

### 4.2. Accuracy of Kinematic Postural Parameters Estimation

Considering the accuracy in the estimation of the kinematic postural parameters, the SDI method showed better performance in tasks with lower CoM displacement, while the BMI method achieved better results in more dynamic tasks. However, the correlation analysis unveiled a stronger Pearson’s correlation coefficients between the BMI-based and the OS-based parameters, when compared to the correlation coefficients between the SDI-based and the OS-based parameters, for all analyzed tasks. We can speculate that this different behavior could be related to the different weights of fixed and random error in the two methods—predominantly fixed in the BM, while predominantly random in the SDI. Deeper and more specific analyses should be performed to accurately explore this topic. Moreover, it would be of interest to evaluate if the application of methods based on machine learning techniques, as already proposed by some authors in this field [42,54,55], could enhance the accuracy of the CoM trajectory estimation, or reduce the number of sensors to be used in the biomechanical model. Future studies should be focused on these research areas.

### 4.3. Limits

A limit of the study is the relatively small number of subjects involved; although comparable or higher than those enrolled in similar validation studies [5,35,38,41]. Moreover, we only investigated healthy subjects, and therefore, further studies should be addressed to evaluate the feasibility of the methods in different samples, especially in patients with neurological or orthopedic diseases.

In addition, the following limitations should be considered. (a) The tests were carried out in an environment free of magnetic interferences, but this condition might not always be possible outside of the laboratory. (b) The considered postural tasks did not actually replicate all standard tasks in targeted applications, such as sport, ergonomics, clinical practice, and therefore, additional tests should be carried out for more demanding tasks; (c) To actually evaluate the possible ecological use of IMUs outside the laboratory, further tests should be carried out evaluating the accuracy in movements performed for a long period of time. (d) The performances of the tested algorithms could be influenced by several factors such as different type of sensors; drift, noise and temperature influence; and, different orientation algorithms, which were not evaluated in this analysis. (e) The lack of the use of a full-body marker set to obtain a more accurate estimation of the CoM trajectory. Future investigations should be considered to address the role of these aspects on the accuracy of the presented methods.

## 5. Conclusions

Our findings showed that the estimation of CoM displacement, based on MIMU, is reasonably accurate, with the mean RMSE values for the three directions ranging from 0.3 to 79.5 mm for the SDI methods, and from 0.9 to 26.2 mm for the BM, therefore, MIMUs could be used for a more ecological evaluation of posture and balance performance, to investigate postural control in athletes, or in patients with orthopedic or neurological diseases, as well as their changes over time, due to the natural course or to the effects of a pharmacological or a rehabilitation intervention. Moreover, our results suggest that, when possible, the use of the inertial sensors network methods should be preferred over a strapdown integration method, based on a single sensor to estimate kinematic parameters.

## Figures and Tables

**Table 1:** Root mean square errors (RMSEs) of the two methods based on MIMUs (Biomechanical Model, BM and Strapdown Integration, SDI), calculated using the optoelectronic system as the reference method, for the six considered tasks. RMSEs are computed for each component of the CoM—anteroposterior (AP), mediolateral (ML), and vertical (V), as well as separately for the modulus (mod). Negative RMSE differences refer to lower RMSEs in the BM; positive RMSE differences cells refer to lower RMSEs in the SDI. P values refer to the paired *t*-tests.

Task	Component	BM RMSE		SDI RMSE		Difference	*p*-Value
		*Mean (SD)*	*Range*	*Mean (SD)*	*Range*	*Mean (SD)*	
**Double Leg Stance**	AP (mm)	6.5 (3.0)	1.8–19.8	6.1 (1.8)	3.3–13.2	0.4 (3.6)	0.514
	ML (mm)	2.5 (0.9)	1.2–4.7	1.9 (1.6)	0.9–9.1	0.5 (1.4)	0.045
	V (mm)	0.9 (0.3)	0.5–1.6	0.3 (0.1)	0.2–0.8	0.6 (0.3)	<0.001
	mod (mm)	5.4 (2.6)	3.5–18.0	4.5 (1.3)	3.2–10.2	0.9 (3.1)	0.129
**Single-Leg Stance**	AP (mm)	26.2 (15.1)	5.6–63.9	11.0 (5.0)	4.6–29.3	15.2 (15.1)	<0.001
	ML (mm)	12.0 (10.1)	0.0–42.7	8.7 (8.7)	2.6–36.3	3.8 (10.6)	0.066
	V (mm)	4.0 (3.1)	1.1–13.9	2.0 (1.4)	0.8–6.5	2.0 (3.5)	0.005
	mod (mm)	23.8 (19.3)	3.3–68.7	9.8 (7.2)	3.8–35.4	14.0 (21.0)	0.001
**AP sway**	AP (mm)	18.9 (7.5)	9.1–38.2	32.0 (17.6)	15.8–90.8	−13.1 (17.0)	<0.001
	ML (mm)	7.6 (2.3)	4.5–12.4	6.1 (2.2)	3.2–14.9	1.4 (2.5)	0.004
	V (mm)	8.9 (2.5)	4.5–12.8	5.5 (3.1)	1.5–14.5	3.3 (3.5)	<0.001
	mod (mm)	17.1 (5.6)	9.2–29.8	24.5 (4.2)	13.1–31.8	−7.4 (4.9)	<0.001
**ML sway**	AP (mm)	6.3 (1.3)	4.6–9.1	10.5 (3.6)	4.8–17.8	−4.1 (3.8)	<0.001
	ML (mm)	23.9 (25.2)	6.8–133.3	48.6 (42.2)	13.4–171.4	−24.7 (33.0)	<0.001
	V (mm)	8.4 (4.1)	2.5–16.6	3.0 (1.6)	1.3–8.8	5.3 (3.9)	<0.001
	mod (mm)	18.4 (11.7)	6.2–44.7	36.9 (14.2)	13.7–72.5	−18.5 (14.2)	<0.001
**Free sway**	AP (mm)	15.8 (7.8)	5.4–34.8	26.9 (17.7)	10.6–84.7	−11.1 (15.9)	0.001
	ML (mm)	23.9 (14.2)	7.2–59.3	44.1 (26.8)	16.2–124.3	−20.2 (23.2)	<0.001
	V (mm)	11.4 (4.6)	3.7–20.5	4.9 (2.6)	1.8–12.8	6.5 (4.2)	<0.001
	mod (mm)	18.5 (9.3)	7.7–44.5	35.3 (10.3)	21.8–56.9	−16.8 (11.3)	<0.001
**Squat**	AP (mm)	17.0 (10.9)	4.2–52.4	41.9 (17.4)	7.3–72.6	−24.9 (19.0)	<0.001
	ML (mm)	9.7 (5.0)	3.4–23.5	6.5 (2.6)	2.1–13.0	3.3 (5.7)	0.004
	V (mm)	24.4 (11.4)	5.9–48.5	79.5 (30.3)	33.5–153.7	−55.1 (28.2)	<0.001
	mod (mm)	22.3 (9.7)	6.3–43.9	84.7 (32.0)	41.8–159.5	−62.4 (29.8)	<0.001

**Table 2:** Pearson’s correlation coefficients between the kinematic parameters computed using each method, based on inertial sensors (Biomechanical Model based on an Inertial Sensors Network, BM, and Strapdown Integration, SDI) and the optoelectronic system (OS). AP—anteroposterior, ML—mediolateral. Pearson’s correlation coefficient interpretation: 0.0–0.2 very weak correlation; 0.2–0.4 weak correlation; 0.4–0.7 moderate correlation; 0.7–1.0 strong correlation.

Task	Kinematic Parameter	Pearson’s Correlation Coefficient (*p* Value) between OS and BM	Pearson’s Correlation Coefficient (*p* Value) between OS and SDI
**Double Leg Stance**	AP Sway	0.579 (0.001)	0.238 (0.204)
	ML Sway	0.779 (<0.001)	0.466 (0.009)
	95% Sway Area	0.331 (0.074)	0.154 (0.418)
	Mean Sway Velocity	0.838 (<0.001)	0.514 (0.004)
**Single Leg Stance**	AP Sway	0.692 (<0.001)	0.501 (0.006)
	ML Sway	0.892 (<0.001)	0.806 (<0.001)
	95% Sway Area	0.904 (0.001)	0.811 (0.552)
	Mean Sway Velocity	0.869 (<0.001)	0.922 (<0.001)
**AP Sway**	AP Sway	0.831 (<0.001)	0.688 (<0.001)
	ML Sway	0.699 (<0.001)	0.287 (0.124)
	95% Sway Area	0.703 (<0.001)	0.557 (0.001)
	Mean Sway Velocity	0.942 (<0.001)	0.962 (<0.001)
**ML Sway**	AP Sway	0.822 (<0.001)	0.416 (0.025)
	ML Sway	0.930 (<0.001)	0.630 (<0.001)
	95% Sway Area	0.810 (<0.001)	0.535 (0.003)
	Mean Sway Velocity	0.935 (<0.001)	0.873 (<0.001)
**Free Sway**	AP Sway	0.919 (<0.001)	0.806 (<0.001)
	ML Sway	0.962 (<0.001)	0.521 (0.003)
	95% Sway Area	0.946 (<0.001)	0.708 (<0.001)
	Mean Sway Velocity	0.980 (<0.001)	0.873 (<0.001)
**Squat**	AP Sway	0.917 (<0.001)	0.437 (0.016)
	ML Sway	0.586 (0.001)	0.293 (0.116)
	95% Sway Area	0.677 (<0.001)	0.388 (0.034)
	Mean Sway Velocity	0.933 (<0.001)	0.675 (<0.001)
